# The Efficacy of Hyperbaric Oxygen Therapy on Middle Cerebral Artery Occlusion in Animal Studies: A Meta-Analysis

**DOI:** 10.1371/journal.pone.0148324

**Published:** 2016-02-09

**Authors:** Yang Xu, Renjie Ji, Ruili Wei, Bo Yin, Fangping He, Benyan Luo

**Affiliations:** 1 Department of Neurology, Brain Medical Centre, First Affiliated Hospital, Zhejiang University School of Medicine, Hangzhou, China; 2 Department of Neurology, Renmin Hospital of Wuhan University, Wuhan, China; Federico II University of Naples, ITALY

## Abstract

**Background:**

Inconsistent results have been reported for hyperbaric oxygen therapy (HBO) for acute stroke. We conducted a systematic review and meta-analysis to evaluate the benefit of HBO in animal studies of middle cerebral artery occlusion (MCAO).

**Methods:**

A systematic search of the literature published prior to September 2015 was performed using Embase, Medline (OvidSP), Web of Science and PubMed. Keywords included “hyperoxia” OR “hyperbaric oxygen” OR “HBO” AND “isch(a)emia” OR “focal cerebral ischemia” OR “stroke” OR “infarct” OR “middle cerebral artery occlusion (MCAO).” The primary endpoints were the infarct size and/or neurological outcome score evaluated after HBO treatment in MCAO. Heterogeneity was analyzed using Cochrane Library’s RevMan 5.3.5.

**Results:**

Fifty-one studies that met the inclusion criteria were identified among the 1198 studies examined. When compared with control group data, HBO therapy resulted in infarct size reduction or improved neurological function (32% decrease in infarct size; 95% confidence interval (CI), range 28%–37%; *p* < 0.00001). Mortality was 18.4% in the HBO group and 26.7% in the control group (RR 0.72, 95% CI, 0.54–0.98; *p* = 0.03). Subgroup analysis showed that a maximal neuro-protective effect was reached when HBO was administered immediately after MCAO with an absolute atmospheric pressure (ATA) of 2.0 (50% decrease; 95% CI, 43% -57% decrease; *p* < 0.0001) and more than 6 hours HBO treatment (53% decrease; 95% CI, 41% -64% decrease; *p* = 0.0005).

**Conclusions:**

HBO had a neuro-protective effect and improved survival in animal models of MCAO, especially in animals given more than 6 hours of HBO and when given immediately after MCAO with 2.0 ATA.

## Introduction

Acute stroke remains a serious issue worldwide [1], especially in low-to middle-income countries [2]. Cerebral hypoxia causes immediate and secondary cell damage, leading disability or even death. Hyperbaric oxygen therapy (HBO) increases oxygen supply to ischemic tissues and reduces irreversible tissue damage. However, the use of HBO for the treatment of ischemic stroke in the clinic remains controversial [3].

During HBO therapy, a patient breathes 100% oxygen in a pressurized environment of at least 1.4 absolute atmospheres (ATA) [4]. A typical treatment consists of oxygen given at 1.5 to 3.0 ATA for 90 to 120 minutes [5]. The results of recent clinical trials have indicated that HBO might improve neurological outcome in post stroke patients, and that this improvement might be seen even when HBO is administered during chronic late stages [6, 7]. However, other studies have reported no beneficial effects following HBO therapy in post stroke patients[8]. This conflict has been mirrored in the results reported by studies examining HBO in animal models of stroke [9, 10]. The conflict in the literature may be the result of study specific variations in initiation time, treatment duration and HBO pressure.

To address the controversy surrounding the efficacy of HBO for cerebral ischemia treatment, and to investigate variations in HBO efficacy under different experimental conditions, we performed a systematic review and meta-analysis of studies that investigated HBO therapy in animal models of middle cerebral artery occlusion (MCAO).

## Materials and Methods

### Database and Literature Search Strategies

A systematic search of the literature from January 1960 to September 2015 was conducted using PubMed, Embase, Medline (OvidSP) and Web of Science. The keywords used for the search were “hyperoxia” OR “hyperbaric oxygen” OR “HBO” AND “isch(a)emia” OR “focal cerebral ischemia” OR “stroke” OR “infarct” OR “middle cerebral artery occlusion (MCAO).”

### Study selection and inclusion criteria

Studies were independently screened by two investigators (Yang Xu and Ruili Wei). Study inclusion criteria were: 1) HBO was used to treat ischemic stroke induced by transient MCAO, embolic MCAO or permanent MCAO; 2) Study design included a control group; and 3) Neuro-behavior scores, infarct size and mortality were compared between groups. Study exclusion criteria were: 1) Not a primary study; 2) Not an animal study; 3) Not a study of the disease of interest (MCAO); and 4) HBO therapy was not used. A third investigator (Bo Yin) resolved any disagreements between the primary screening investigators. The primary endpoints examined were infarct area or volume (as determined by 2, 3, 5-triphenyltetrazolium chloride (TTC) staining of tissue sections or cross-sectional MRI imaging) and neuro-behavior scores.

### Study characteristics

The quality of the evidence in each study was assessed using the following criteria [11]: A) publication after peer review, B) inclusion of a statement concerning temperature control, C) random allocation to treatment or control groups, D) masked induction of ischemia, E) masked assessment of outcome, F) use of anesthetic without significant intrinsic neuroprotective activity, G) use of an appropriate animal model (aged, diabetic or hypertensive), H) appropriate sample size calculation, I) compliance with animal welfare regulations and J) the presence of an appropriate statement of potential conflict of interests. The maximum total score for any study was ten points.

### Data Extraction

**Fig 1:**
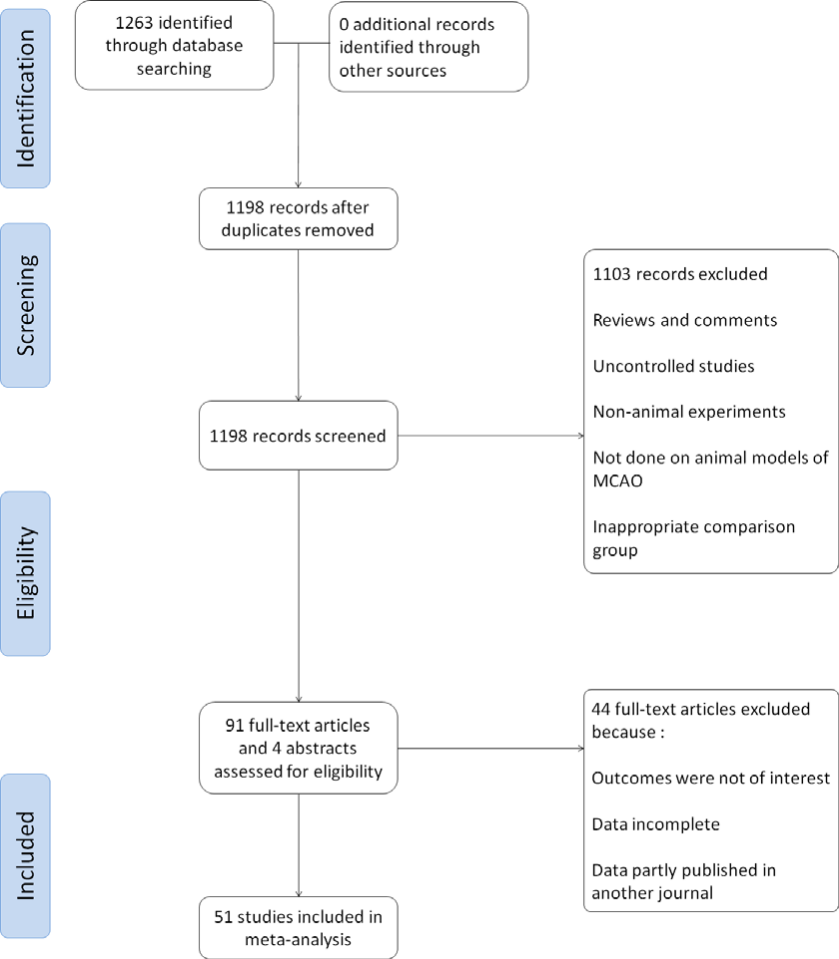
Flow diagram of study selection process.

Data was independently extracted by two investigators according to the inclusion criteria described above (Fig 1). Comparisons were performed between the HBO treatment and control groups of the selected studies. For each comparison, the mean outcome, standard deviation and number of animals in each group were examined. When data was found to be missing, the original authors of the study in question were contacted for additional information. If data were only represented graphically, numerical values were requested from the original authors. If no response was received to requests for numerical data, digital scale software Image J (National Institutes of Health, Bethesda, MD, USA) was utilized to extract numerical data from the available graphical data. We used the ‘#’ symbol to mark these articles, and they were presented in Table 1. Effect size was defined as the improvement in infarct size, neurologic score or combined score in treated animals when compared with the control groups. Other data collected included the timing of treatment initiation (preconditioning before ischemia, treatment concurrent with an ischemic event or treatment after ischemia and reperfusion), duration of HBO, oxygen pressure, ischemia induction method, combined drug therapy and animal morality rate.

**Table 1:** Characteristics of the animal studies included in the meta-analysis.

Author	Year	Species	Stroke model	Time of ischemia	Time from onset	HBO treatment	Length	Outcome Measures	Quality score
W. J. Yan # [12]	2011	SD	tMCAO	120 min	5 d before occlusion	100% O_2_ 2.5 ATA	1 h*5	Infarct Volume	5
Kuppers-Tiedt [13]	2011	Wistar	eMCAO		45 min or 120 min after occlusion	100% O_2_ 2.4 ATA	1 h	Combined	6
Mu [14]	2013	SD	pMCAO		48 h after occlusion	100% O_2_ 2.5 ATA	1 h*10	Infarct Volume	6
Badr [10]	2001	SD	tMCAO	120 min	3 h, 6 h, 12h, 23h after occlusion	100% O_2_ 3 ATA	1 h	Combined	5
Beynon [15]	2007	Wistar	tMCAO	150 min	90 min or 120 min after occlusion	100% O_2_ 3 ATA	1 h*1	Combined	6
Wang [16]	2012	SD	tMCAO	60 min	3 h before occlusion or 3 h after reperfusion	100% O_2_ 3 ATA	1 h*1	Infarct Volume	4
Veltkamp [17]	2000	Wistar	tMCAO	60 min	Immediately after occlusion	100% O_2_ 1.5 ATA or 100% O_2_ 2.5 ATA	2 h*1	Infarct Volume	6
Lu # [9]	2014	Wistar	tMCAO	90 min	3 h after reperfusion	100% O_2_ 3 ATA	1 h*1	Infarct Volume	5
Bian [18]	2015	SD	tMCAO	120 min	5 d before occlusion	100% O_2_ 2.5 ATA	1 h*5	Combined	6
Soejima [19]	2012	2H	tMCAO	120 min	5 d before occlusion	100% O_2_ 2.5 ATA	1 h*5	Combined	6
Soejima # [20]	2013	SD	tMCAO	90 min	5 d before occlusion	100% O_2_ 2.5 ATA	1 h*5	Combined	6
Li [21]	2009	SD	tMCAO	90 min	4 d before occlusion	100% O_2_ 2.5 ATA	1 h*4	Combined	6
Chang [22]	2000	SD	tMCAO	60 min	Immediately or 1 h after occlusion	100% O_2_ 3 ATA	1.5 h*2	Infarct Volume	6
Prass [23]	2000	SV129	tMCAO	60 min	5 d before occlusion	100% O_2_ 3 ATA	1 h*5	Infarct Volume	5
Qin [24]	2007	SD	tMCAO	60 min	0.5 h after occlusion	100% O_2_ 3 ATA	1 h*1	Combined	6
Yin [25]	2003	SD	tMCAO	120 min	6 h after occlusion	100% O_2_ 2.5 ATA	2 h*1	Infarct Volume	5
Lou [26]	2006	SD	tMCAO	90 min	1.5 h after occlusion	100% O_2_ 3 ATA	1 h*1	Infarct Volume	6
Li [27]	2008	SD	pMCAO		24 h before occlusion	100% O_2_ 2.5 ATA	2.5 h*1	Infarct Volume	3
Gu [28]	2008	SD	pMCAO		5 d before occlusion	100% O_2_ 2 ATA	1 h*5	Behavioral Score	5
Schabitz [29]	2004	Wistar	pMCAO		2 h after occlusion	100% O_2_ 2 ATA	1 h*1	Combined	5
Eschenfelder [30]	2008	SD	tMCAO	90 min	1.5 h after reperfusion	100% O_2_ 2 ATA, 2.5 ATA or 3 ATA	1.5 h*1	Combined	6
Miljkovic-Lolic [31]	2003	SD	tMCAO	60 min	Immediately or 1 h before occlusion	100% O_2_ 3 ATA	1 h*1	Combined	5
Sun [32]	2014	C57	tMCAO	90 min	15 min after occlusion	100% O_2_ 3 ATA	1 h*1	Infarct Volume	5
Sun [33]	2011	C57	tMCAO	90 min	15 min after occlusion	100% O_2_ 3 ATA	1 h*1	Infarct Volume	6
Veltkamp [34]	2006	C57	tMCAO	90 min	45 min after occlusion or 1 d, 3 d, 5 d after reperfusion	100% O_2_ 3 ATA	1 h*1	Infarct Volume	6
Rink # [35]	2010	Wistar	tMCAO	90 min	Immediately after occlusion or reperfusion	100% O_2_ 2 ATA	1.5 h*1	Infarct Volume	5
Gunther [36]	2005	2H	pMCAO		0.5 h to 6 h after occlusion	100% O_2_ 2.5 ATA	1.5 h*1, 1.5 h*2 or 1.5 h*4	Infarct Volume	6
Chen # [37]	2014	SD	tMCAO	90 min	1.5 h after reperfusion	100% O_2_ 2 ATA	1 h*2, 1 h*4, 1 h*6, or 1 h*8	Infarct Volume	6
Weinstein [38]	1987	Cat	tMCAO	6 h	1 h, 3 h or 4 h after occlusion	100% O_2_ 1.5 ATA	0.75 h*1	Infarct Volume	3
Yan # [39]	2013	SD	tMCAO	120 min	5 d before occlusion	100% O_2_ 2.5 ATA	1 h*5	Infarct Volume	4
Liu [40]	2010	SD	tMCAO	90 min	1.5 h after occlusion	100% O_2_ 3 ATA	1 h*1	Infarct Volume	6
Jiang [41]	2006	SD	tMCAO	-	-	-	-	Infarct Volume	3
Hou [42]	2007	SD	tMCAO	120 min	0.5 h after occlusion or 1.5 h after reperfusion	100% O_2_ 2 ATA	1 h*1	Infarct Volume	5
Huang [43]	2007	SD	eMCAO		1 h after occlusion	100% O_2_ 2.5 ATA	2 h*1	Combined	5
Kawamura [44]	1990	SD	tMCAO	4 h	4 h after occlusion	-	3.5 h*1	Infarct Volume	4
Lou [45]	2004	SD	tMCAO or pMCAO	1.5 h	4 h, 6 h or 12 h after occlusion	100% O_2_ 3 ATA	1 h*1	Infarct Volume	6
Yin [46]	2005	SD	tMCAO	2 h	6 h or 24 h after reperfusion	100% O_2_ 2.5 ATA	2 h*7 or 2 h*6	Infarct Volume	5
Xue # [47]	2008	SD	tMCAO	1.5 h	3 h after occlusion	100% O_2_ 2 ATA	9 h*1 or 18 h*1	Infarct Volume	5
Xiong [48]	2000	SD	tMCAO	1.5 h	5 d before occlusion	100% O_2_ 2.5 ATA	1 h*3 or 1 h*5	Combined	5
Sunami [49]	2000	SD	tMCAO	2 h	10 min after occlusion	100% O_2_ 3 ATA	2 h*1	Infarct Volume	5
Harman [50]	2012	SD	pMCAO		before occlusion	100% O_2_ 2.8 ATA	0.75 h*1	Infarct Volume	4
Acka [51]	2007	Wistar	tMCAO	1.5h	Immediately after occlusion	100% O_2_ 2.5 ATA	1.5 h*1	Infarct Volume	6
Yin # [52]	2002	SD	tMCAO	2 h	8 h after occlusion	100% O_2_ 3 ATA	1 h*1	Infarct Volume	5
Badr # [53].	2001	SD	tMCAO	2 h	8 h after occlusion	100% O_2_ 3 ATA	1 h*1	Infarct Volume	4
Veltkamp [54]	2005	Wistar	tMCAO	2 h	0.5 h after occlusion	100% O_2_ 3 ATA	1 h*1	Infarct Volume	4
Li [55]	2008	SD	tMCAO	2 h	1 d before occlusion	100% O_2_ 2.5 ATA	1 h*4	Behavioral Score	5
Veltkamp [56]	2005	Wistar	tMCAO	2 h	0.5 h after occlusion	100% O_2_ 3 ATA	1 h*1	Infarct Volume	5
Sun [57]	2008	C57	tMCAO	2 h	0.5 h after occlusion	100% O_2_ 3 ATA	1.5 h*1	Infarct Volume	6
Lou [58]	2007	SD	pMCAO		1 h after occlusion	100% O_2_ 3 ATA	1 h*1	Combined	6
Henninger [59]	2006	Wistar	eMCAO		3 h after occlusion	100% O_2_ 2.5 ATA	1 h*1	Infarct Volume	6
Yang [60]	2010	SD	tMCAO	1h	Immediately after occlusion	100% O_2_ 2.8 ATA	0.25 h*4	Infarct Volume	5

# showed the study in which Image J (NIH) software was used to extract data from images.

* means HBO treatment duration and frequency

### Statistical Analysis

For primary outcome comparisons (infarct volumes and neuro-behavior scores), the mean outcomes of treatment groups and the standard deviations of treatment and control groups were expressed as a proportion of control group outcomes. The proportion of the mean values of the HBO groups to the mean values of the control groups was used as a new parameter. Data analysis was conducted using weighted mean differences and a random effects model. To explore the impact of various parameters on estimates of effect size, we performed a stratified meta-analysis using the following parameters: timing of treatment initiation, treatment duration, ischemia induction method, oxygen pressure, combined drug therapy, continuous or intermittent HBO treatment and species of animal used. *p* < 0.01 was considered significant for multiple comparisons. Forest Plots and funnel plots were generated using RevMan 5.3.5 software (Cochrane Library, London, UK).

## Results

### Study Inclusion and Characteristics

We identified 51 publications describing the effect of HBO in focal cerebral ischemia that met our inclusion criteria (Table 1). These publications included 47 full articles and 4 abstracts. The earliest study included was published in 1987. Among these studies, 111 comparisons were identified describing outcomes in 2456 animals (all included animals were male). Rat MCAO models (Wistar or Sprague-Dawley rats) were used in 42 studies, 5 studies used mice (C57 or SV129), 3 studies used hyperglycemia or hypertension rats (2H) rats and 1 study was performed in cats. Forty of these studies utilized a transient MCAO (tMCAO) animal model, seven used permanent MCAO (pMCAO), three used Endothelin-1 or autologous blood clot embolic MCAO (eMCAO) and one used both tMCAO and pMCAO. Thirty-five publications reported infarct volume and 14 reported neuro-behavior scores with infarct volume. Two publications only reported neuro-behavior outcomes. Thirteen publications provided morality rate. None of the included studies addressed side effects. Treatment initiation ranged from five days before to two days after ischemia induction. Effect size was measured at a median of 24 hours (6 hours to 28 days) after ischemia onset.

### Efficacy

**Fig 2:**
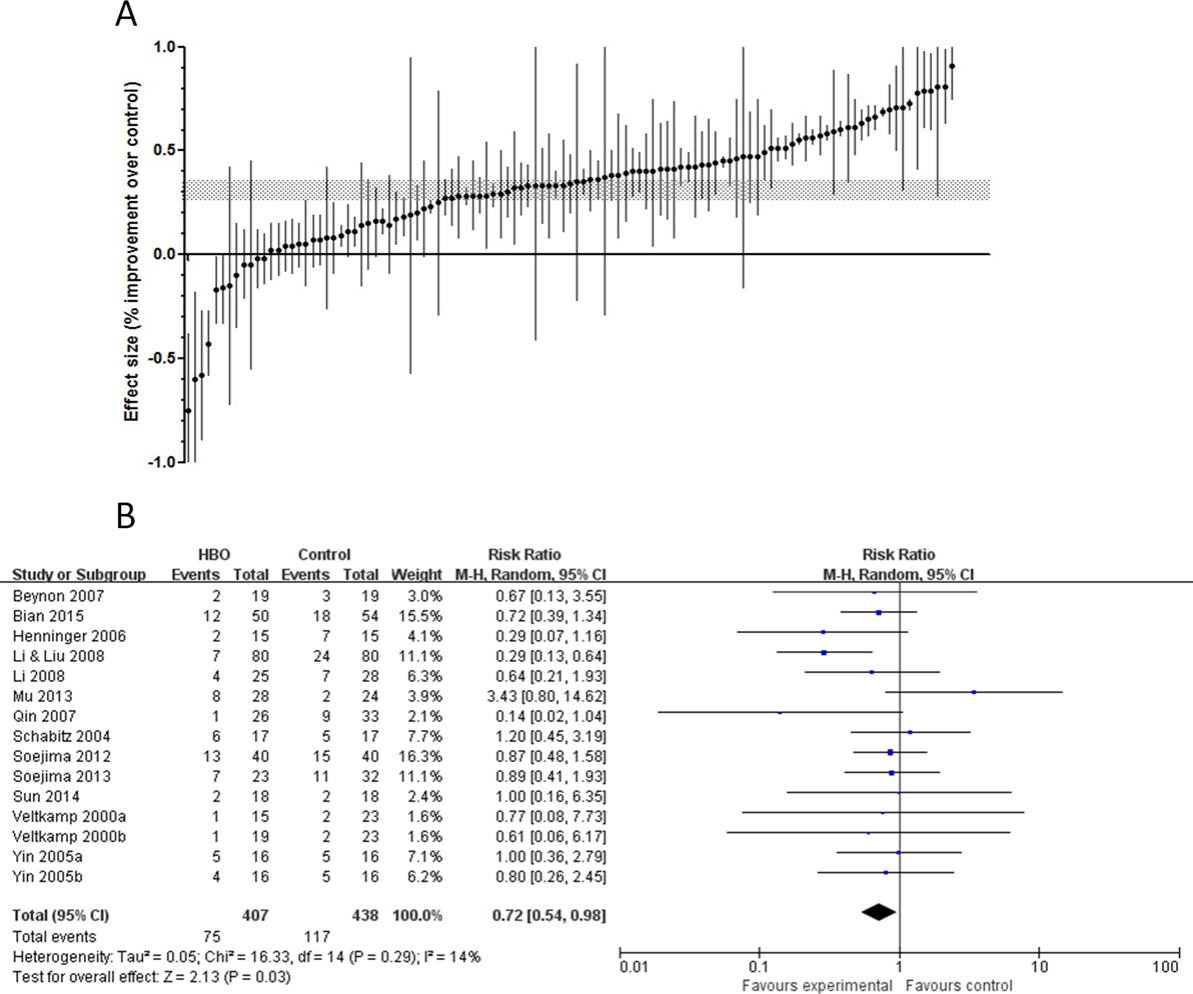
Comparisons ranked according to effect size and comparison of mortality rate. (A) The shaded gray band represents the 95% CI for the global estimate. The vertical error bars represent the 95% CI for individual comparisons. (B) Forest plot of comparison: HBO *vs* Control, outcome: Mortality.

HBO treatment decreased infarct volume by 32% (95% confidence interval (CI), 28%–37% decrease; *p* < 0.00001) with significant heterogeneity (χ2 = 3345.29, df = 110, *p* < 0.00001, *I*^2^ = 97%; Fig 2A). The mortality was 18.4% in the HBO group and 26.7% in the control group (Risk Ratio (RR) 0.72, 95% CI, 0.54–0.98, *p* = 0.03; Fig 2B).

### Subgroup analysis

**Fig 3:**
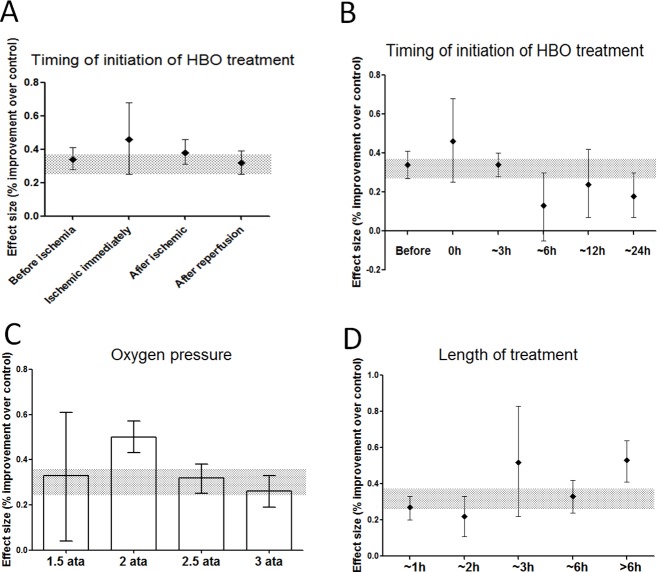
Point estimates and 95% CIs of effect size by: (A,B) Timing of initiation of HBO treatment, (C) Oxygen pressure, (D) Length of treatment. The 95% CI for the global estimate is shown as a grey band.

Treatment initiation ranged from five days before to two days after the onset of ischemia. All treatment initiation subtypes (preconditioning before ischemia, concurrent, post treatment after ischemia and reperfusion) exhibited protective effects (Fig 3A). The smallest infarct size and best neurologic function was observed when HBO was administered immediately after MCAO (Fig 3B). However, HBO reduced infarct volume even when it was initiated 12 hours after ischemia.

Significant neuro-protective effects were observed at all oxygen pressures used in the selected studies (pressures ranged from 1.5 ATA to 3.0 ATA; Fig 3C). However, the maximum neuro-protective effect was observed near 2.0 ATA (50% decrease; 95% CI; range, 43% to 57% decrease; χ2 = 23.01; df = 3; *p* < 0.0001).

Protective effects were found for all treatment durations in the selected studies (ranging from 45 minutes to 18 hours), with a maximum effect following approximately 6 hours of HBO treatment (53% decrease; 95% CI; range, 64% decrease to 41% increase; χ2 = 19.51; df = 4; *p* = 0.0006; Fig 3D).

**Fig 4:**
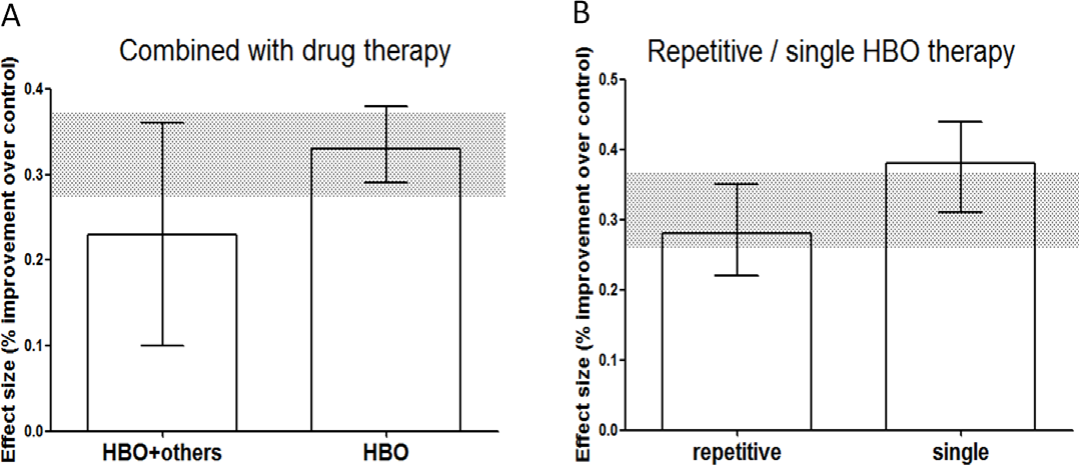
Point estimates and 95% CIs of effect size by: (A) HBO combined with drug therapy, (B) Repetitive or single HBO therapy. The 95% CI for the global estimate is shown as a grey band.

No statistically significant difference was observed between HBO treatment alone and HBO treatment given in combination with any drug in the selected studies (Fig 4A). Repetitive oxygen therapy trended towards greater efficacy when compared with single oxygen therapy; however, this trend was not significant (χ2 = 3.90, df = 1, *p* = 0.05; Fig 4B).

**Fig 5:**
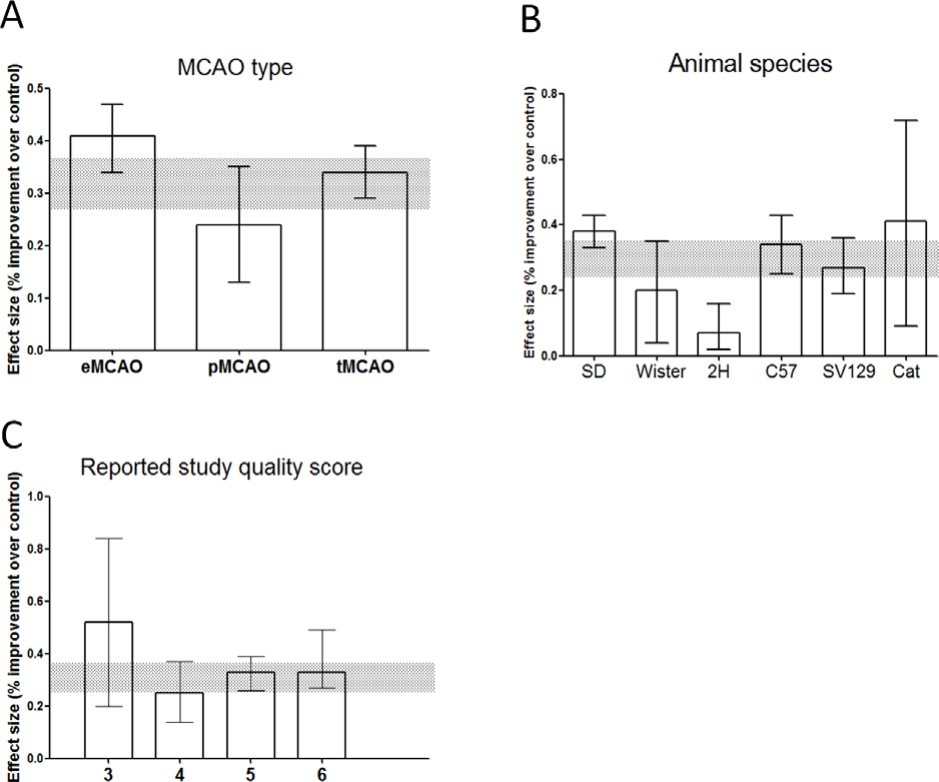
Point estimates and 95% CIs of effect size by: (A) MCAO type, (B) Animal species, (C) Reported study quality score. The 95% CI for the global estimate is shown as a grey band.

Different types of MCAO (transient or permanent) had little effect on evaluation results (χ^2^ = 5.38, df = 2, *p* = 0.05; Fig 5A). Subgroup analysis showed HBO effects on different animal species might not be the same. The reduced infarct volumes in the SD and cat groups were more significant than the volume reductions observed in the other groups, and were minimal in the 2H group (χ^2^ = 27.88, df = 5, *p* < 0.0001; Fig 5B).

### Study Quality and Publication Bias

None of the included studies reported statements concerning potential conflicts of interest, sample size calculation or masked induction of ischemia. Thirty-eight of the included studies used random allocation of animals to treatment groups. Only two studies used animal models relevant to specific clinical situations (e.g., hyperglycemia or hypertension) [18, 36]. The median included quality score was 5 (range 3 to 6). Although no significant differences were observed when studies were ranked by quality score (Fig 5C), this still represents an important potential source of bias. The funnel plot revealed an asymmetrical distribution of studies around the line of identity (S1 Fig).

## Discussion

This meta-analysis revealed that HBO therapy provoked a significant neuro-protective effect and improved survival in animal models of MCAO. Maximum efficacy was achieved when intervention was implemented immediately after brain ischemia/reperfusion and maintained for more than 6 hours at 2.0 ATA.

The MCAO model is frequently used to imitate acute stroke. Ischemic stroke is induced by arterial occlusion or arterial stenosis. During ischemia, decreased cerebral blood flow reduces the availability of oxygen and glucose, leading to excitatory toxicity, depolarization, inflammation, and the production of nitric oxide (NO), reactive oxygen species and toxic prostanoids [61]. An ischemic core with a surrounding penumbra is formed when the blood flow to surrounding regions is impaired [62]. This is a temporal phenomenon and rarely seen in the clinic. The current review observed that HBO was effective even when HBO was administered 12 hours after the onset of ischemia. These results indicate that the therapeutic window could be expanded. This is a particularly striking result because it indicates that the currently accepted time window for thrombolysis in the clinic of 3 to 6 hours might be too conservative [63]. All HBO treatment dosages were associated with improved neurological function, however, treatment under 2.0 ATA maximized efficacy in animal studies, which is slightly lower than the 2.5 ATA pressure recommended by the Committee of the Undersea and Hyperbaric Medical Society [14]. The current meta-analysis observes that HBO efficacy improvement begins after 3 hours of treatment and reaches a maximum at 6 hours. This pattern of treatment response might be related to decreased oxygen intoxication and improved HBO tolerance. This hypothesis is supported by a study by Beynon et. al. that reported that HBO efficacy appears to be time-dependent [15]. The current meta-analysis observed no additional efficacy when HBO therapy was combined with other drugs. However, combining HBO with thrombolysis was associated with a trend towards fewer hemorrhagic complications [13]. No efficacy differences were observed between ischemia period and the different MCAO variations (eMCAO, pMCAO or tMCAO).

The mechanism of HBO therapy provoked neuroprotection remains unclear. However, reduced brain edema and improved post-ischemia metabolism seem contribute to the observed beneficial effects. HBO appears to increase the expression of antioxidant enzymes, alleviate oxidative stress [55], reduce the formation of hydroxyl radicals [60], enhance superoxide dismutase and catalase activity [55, 64] and inhibit the activity of nitrogen monoxide synthase (NOS) [65]. Additionally, HBO modulate inflammation in the brain by increasing anti-inflammatory cytokines (such as IL-10) and inhibiting pro-inflammatory cytokines (such as TNF-α and IL-1-β) [37]. Furthermore, HBO might reduce interactions between inflammatory chemokines and infiltrating neutrophils, resulting in restricted leukocyte aggregation in the ischemic-hypoxic area. Moreover, HBO may directly affect cyclooxygenase-2 (COX-2) activity in ischemic hemispheres [52]. Finally, it is likely that inhibition of apoptosis by HBO treatment suppresses mitochondrial apoptotic pathways, reduces cytoplasm cytochrome-c levels, decreases caspase enzyme activity and dysregulates the ratio of B cell lymphoma 2 (Bcl-2) and Bcl-2 associated X (Bax) expression, thereby preventing DNA fragmentation [21, 25].

Interestingly, HBO efficacy might be influenced by the animal species. Subgroup analysis indicated HBO was least effective in the 2H group when compared with other species. This reduction in efficacy might be due to the pathological changes caused by hyperglycemia and hypertension in the 2H group. Because most stroke patients have comorbidities, the 2H group may be the best representation of clinical reality among the animal models examined here. Unfortunately, these confounding factors increase the uncertainty of HBO efficacy.

The beneficial effects of HBO therapy observed in animal models were different from those reported in clinical trials. There may be a number of explanations for these discrepancies. First, patients with ischemic stroke might have a narrow therapeutic time window while this time window is relatively longer in animal experiments. Many clinical patients are treated later after stroke onset and showed no beneficial effects [8, 66, 67]. Second, higher than optimal pressure was used for HBO treatment in the studies examined. Although evidence suggests 2.5 ATA may be optimal for improved outcome and smaller infarctions, we consider 2.0 ATA more appropriate in current meta-analysis. Indeed, Sánchez *et al*. used 1.8 ATA HBO over 6 hours to treat stroke and reported a significant reduction of infarct volume [5]. A clinical trial by Imai *et al*. reported that HBO therapy at 2.0 ATA has been associated with favorable outcomes [68].

Higher pressure HBO exposure is a subject of concern because high pressures may impair blood-brain barrier integrity and induce free radical production and lipid peroxidation [32, 69–71]. The production of hydroxyl radicals may lead to decreased cerebral blood flow and central nervous system oxygen toxicity [72]. Additionally, interrupted autophagy flux might play a role. As a clearance pathway, autophagy degrades dysfunctional proteins after MCAO. Interruption of autophagy could result in dysfunctional protein aggregation and neuronal death [9]. Therefore, it is important to consider key autophagy factors (including autophagy-related molecules (Microtubule-associated protein 1 light chain 3, LC3) and autophagy substrates when investigating potentially harmful treatment routines. Additionally, peroxidation lipid (Malonaldehyde, MDA) and NOX levels can help determine optimal HBO pressures in animal experiments.

### Limitations

This study has a number of limitations that should be considered. First, the study had a high degree of heterogeneity and included a number of different animal models, therapeutic strategies and end points. Second, publication bias might overestimate the efficacy of HBO therapy because it is difficult to publish negative results. Third, subgroup analysis (with its lack of statistical power) is always risky. Fourth, most of these studies were performed in healthy animals, which may not accurately replicate the conditions of clinical stroke patients with morbidity. Fifth, side effects were not studied in these experiments. Sixth, the software facilitated data extraction used for 9 articles might have added unintended bias; however, an independent analysis comparing extracted and original data demonstrated similar HBO effects. Finally, the results of these studies must be interpreted with care because the results of animal studies may not always be clinically relevant.

## Conclusion

This study reports that HBO therapy is associated with a substantial neuroprotective effect and improved survival in animal models of MCAO. This effect was most pronounced when 2.0 ATA HBO therapy was administered for more than 6 hours immediately after MCAO. The oxygen pressure, initiation and length of treatment described in this study should be taken into account to maximize effectiveness when designing future clinical studies.

## Supporting Information

S1 PRISMA Checklist PRISMA checklist. (DOC)

S1 Fig Funnel plot overseeing publication bias of included studies. (TIF)

## References

[1] Edwards DF, Hahn M, Baum C, Dromerick AW. The impact of mild stroke on meaningful activity and life satisfaction. Journal of stroke and cerebrovascular diseases. 2006;15(4):151–7. 17904068 10.1016/j.jstrokecerebrovasdis.2006.04.001

[2] O'Donnell MJ, Xavier D, Liu L, Zhang H, Chin SL, Rao-Melacini P, et al Risk factors for ischaemic and intracerebral haemorrhagic stroke in 22 countries (the INTERSTROKE study): a case-control study. Lancet. 2010;376(9735):112–23. 10.1016/S0140-6736(10)60834-3 20561675

[3] Carson S, McDonagh M, Russman B, Helfand M. Hyperbaric oxygen therapy for stroke: a systematic review of the evidence. Clinical rehabilitation. 2005;19(8):819–33. 16323381 10.1191/0269215505cr907oa

[4] Garcia-Covarrubias L, Cuauhtemoc Sanchez-Rodriguez E. Hyperbaric oxygenation therapy, basic concepts. Gaceta medica de Mexico. 2000;136(1):45–56. 10721602

[5] Sanchez EC. Mechanisms of action of hyperbaric oxygenation in stroke: a review. Critical care nursing quarterly. 2013;36(3):290–8. 10.1097/CNQ.0b013e318294e9e3 23736668

[6] Efrati S, Fishlev G, Bechor Y, Volkov O, Bergan J, Kliakhandler K, et al Hyperbaric oxygen induces late neuroplasticity in post stroke patients—randomized, prospective trial. PloS one. 2013;8(1):e53716 10.1371/journal.pone.0053716 23335971 PMC3546039

[7] Chen CH, Chen SY, Wang V, Chen CC, Wang KC, Chen CH, et al Effects of repetitive hyperbaric oxygen treatment in patients with acute cerebral infarction: a pilot study. The Scientific World Journal. 2012;2012:694703 10.1100/2012/694703 22919348 PMC3415162

[8] Rusyniak DE, Kirk MA, May JD, Kao LW, Brizendine EJ, Welch JL, et al Hyperbaric oxygen therapy in acute ischemic stroke: results of the Hyperbaric Oxygen in Acute Ischemic Stroke Trial Pilot Study. Stroke. 2003;34(2):571–4. 12574578 10.1161/01.str.0000050644.48393.d0

[9] Lu YX, Kang JS, Bai Y, Zhang Y, Li HY, Yang XC, et al Hyperbaric oxygen enlarges the area of brain damage in MCAO rats by blocking autophagy via ERK1/2 activation. European journal of pharmacology. 2014;728:93–9. 10.1016/j.ejphar.2014.01.066 24512724

[10] Badr AE, Yin W, Mychaskiw G, Zhang JH. Dual effect of HBO on cerebral infarction in MCAO rats. Am J Physiol-Reg I. 2001;280(3):R766–R70. 10.1152/ajpregu.2001.280.3.R766 11171656

[11] Sena E, Wheble P, Sandercock P, Macleod M. Systematic review and meta-analysis of the efficacy of tirilazad in experimental stroke. Stroke. 2007;38(2):388–94. 17204689 10.1161/01.STR.0000254462.75851.22

[12] Yan WJ, Zhang HP, Bai XG, Lu Y, Dong HL, Xiong LZ. Autophagy activation is involved in neuroprotection induced by hyperbaric oxygen preconditioning against focal cerebral ischemia in rats. Brain research. 2011;1402:109–21. 10.1016/j.brainres.2011.05.049 21684529

[13] Kuppers-Tiedt L, Manaenko A, Michalski D, Guenther A, Hobohm C, Wagner A, et al Combined systemic thrombolysis with alteplase and early hyperbaric oxygen therapy in experimental embolic stroke in rats: relationship to functional outcome and reduction of structural damage. Acta neurochirurgica Supplement. 2011;111:167–72. 10.1007/978-3-7091-0693-8_28 21725750

[14] Mu J, Ostrowski RP, Soejima Y, Rolland WB, Krafft PR, Tang JP, et al Delayed hyperbaric oxygen therapy induces cell proliferation through stabilization of cAMP responsive element binding protein in the rat model of MCAo-induced ischemic brain injury. Neurobiol Dis. 2013;51:133–43. 10.1016/j.nbd.2012.11.003 23146993 PMC3557601

[15] Beynon C, Sun L, Marti HH, Heiland S, Veltkamp R. Delayed hyperbaric oxygenation is more effective than early prolonged normobaric hyperoxia in experimental focal cerebral ischemia. Neuroscience letters. 2007;425(3):141–5. 17850964 10.1016/j.neulet.2007.07.009

[16] Wang RY, Chang HC, Chen CH, Tsai YW, Yang YR. Effects of hyperbaric oxygenation on oxidative stress in acute transient focal cerebral ischemic rats. Eur J Appl Physiol. 2012;112(1):215–21. 10.1007/s00421-011-1976-2 21533807

[17] Veltkamp R, Warner DS, Domoki F, Brinkhous AD, Toole JF, Busija DW. Hyperbaric oxygen decreases infarct size and behavioral deficit after transient focal cerebral ischemia in rats. Brain research. 2000;853(1):68–73. 10627309 10.1016/s0006-8993(99)02250-7

[18] Bian H, Hu Q, Liang X, Chen D, Li B, Tang J, et al Hyperbaric oxygen preconditioning attenuates hemorrhagic transformation through increasing PPARgamma in hyperglycemic MCAO rats. Experimental neurology. 2015;265:22–9. 10.1016/j.expneurol.2014.12.016 25542160 PMC4346496

[19] Soejima Y, Ostrowski RP, Manaenko A, Fujii M, Tang J, Zhang JH. Hyperbaric oxygen preconditioning attenuates hyperglycemia enhanced hemorrhagic transformation after transient MCAO in rats. Medical gas research. 2012;2(1):9 10.1186/2045-9912-2-9 22494892 PMC3351373

[20] Soejima Y, Hu Q, Krafft PR, Fujii M, Tang J, Zhang JH. Hyperbaric oxygen preconditioning attenuates hyperglycemia-enhanced hemorrhagic transformation by inhibiting matrix metalloproteinases in focal cerebral ischemia in rats. Experimental neurology. 2013;247:737–43. 10.1016/j.expneurol.2013.03.019 23537951 PMC3742563

[21] Li JS, Zhang W, Kang ZM, Ding SJ, Liu WW, Zhang JH, et al Hyperbaric oxygen preconditioning reduces ischemia-reperfusion injury by inhibition of apoptosis via mitochondrial pathway in rat brain. Neuroscience. 2009;159(4):1309–15. 10.1016/j.neuroscience.2009.01.011 19185051

[22] Chang CF, Niu KC, Hoffer BJ, Wang Y, Borlongan CV. Hyperbaric oxygen therapy for treatment of postischemic stroke in adult rats. Experimental neurology. 2000;166(2):298–306. 11085895 10.1006/exnr.2000.7506

[23] Prass K, Wiegand F, Schumann P, Ahrens M, Kapinya K, Harms C, et al Hyperbaric oxygenation induced tolerance against focal cerebral ischemia in mice is strain dependent. Brain research. 2000;871(1):146–50. 10882793 10.1016/s0006-8993(00)02264-2

[24] Qin Z, Karabiyikoglu M, Hua Y, Silbergleit R, He Y, Keep RF, et al Hyperbaric oxygen-induced attenuation of hemorrhagic transformation after experimental focal transient cerebral ischemia. Stroke. 2007;38(4):1362–7. 17322079 10.1161/01.STR.0000259660.62865.eb

[25] Yin D, Zhou C, Kusaka I, Calvert JW, Parent AD, Nanda A, et al Inhibition of apoptosis by hyperbaric oxygen in a rat focal cerebral ischemic model. Journal of cerebral blood flow and metabolism. 2003;23(7):855–64. 12843789 10.1097/01.WCB.0000073946.29308.55

[26] Lou M, Chen Y, Ding M, Eschenfelder CC, Deuschl G. Involvement of the mitochondrial ATP-sensitive potassium channel in the neuroprotective effect of hyperbaric oxygenation after cerebral ischemia. Brain research bulletin. 2006;69(2):109–16. 16533658 10.1016/j.brainresbull.2005.11.009

[27] Li Z, Liu W, Kang Z, Lv S, Han C, Yun L, et al Mechanism of hyperbaric oxygen preconditioning in neonatal hypoxia-ischemia rat model. Brain research. 2008;1196:151–6. 10.1016/j.brainres.2007.12.039 18221732

[28] Gu GJ, Li YP, Peng ZY, Xu JJ, Kang ZM, Xu WG, et al Mechanism of ischemic tolerance induced by hyperbaric oxygen preconditioning involves upregulation of hypoxia-inducible factor-1alpha and erythropoietin in rats. Journal of applied physiology. 2008;104(4):1185–91. 10.1152/japplphysiol.00323.2007 18174394

[29] Schabitz WR, Schade H, Heiland S, Kollmar R, Bardutzky J, Henninger N, et al Neuroprotection by hyperbaric oxygenation after experimental focal cerebral ischemia monitored by MRI. Stroke. 2004;35(5):1175–9. 15060313 10.1161/01.STR.0000125868.86298.8e

[30] Eschenfelder CC, Krug R, Yusofi AF, Meyne JK, Herdegen T, Koch A, et al Neuroprotection by oxygen in acute transient focal cerebral ischemia is dose dependent and shows superiority of hyperbaric oxygenation. Cerebrovascular diseases. 2008;25(3):193–201. 10.1159/000113856 18212507

[31] Miljkovic-Lolic M, Silbergleit R, Fiskum G, Rosenthal RE. Neuroprotective effects of hyperbaric oxygen treatment in experimental focal cerebral ischemia are associated with reduced brain leukocyte myeloperoxidase activity. Brain research. 2003;971(1):90–4. 12691841 10.1016/s0006-8993(03)02364-3

[32] Sun L, Wolferts G, Veltkamp R. Oxygen therapy does not increase production and damage induced by reactive oxygen species in focal cerebral ischemia. Neuroscience letters. 2014;577:1–5. 10.1016/j.neulet.2014.05.060 24909618

[33] Sun L, Strelow H, Mies G, Veltkamp R. Oxygen therapy improves energy metabolism in focal cerebral ischemia. Brain research. 2011;1415:103–8. 10.1016/j.brainres.2011.07.064 21872850

[34] Veltkamp R, Sun L, Herrmann O, Wolferts G, Hagmann S, Siebing DA, et al Oxygen therapy in permanent brain ischemia: potential and limitations. Brain research. 2006;1107(1):185–91. 16828721 10.1016/j.brainres.2006.05.108

[35] Rink C, Roy S, Khan M, Ananth P, Kuppusamy P, Sen CK, et al Oxygen-sensitive outcomes and gene expression in acute ischemic stroke. Journal of cerebral blood flow and metabolism. 2010;30(7):1275–87. 10.1038/jcbfm.2010.7 20145654 PMC2913550

[36] Gunther A, Kuppers-Tiedt L, Schneider PM, Kunert I, Berrouschot J, Schneider D, et al Reduced infarct volume and differential effects on glial cell activation after hyperbaric oxygen treatment in rat permanent focal cerebral ischaemia. The European journal of neuroscience. 2005;21(11):3189–94. 15978027 10.1111/j.1460-9568.2005.04151.x

[37] Chen LF, Tian YF, Lin CH, Huang LY, Niu KC, Lin MT. Repetitive hyperbaric oxygen therapy provides better effects on brain inflammation and oxidative damage in rats with focal cerebral ischemia. Journal of the Formosan Medical Association. 2014;113(9):620–8. 10.1016/j.jfma.2014.03.012 24787662

[38] Weinstein PR, Anderson GG, Telles DA. Results of hyperbaric oxygen therapy during temporary middle cerebral artery occlusion in unanesthetized cats. Neurosurgery. 1987;20(4):518–24. 3587541 10.1227/00006123-198704000-00002

[39] Yan W, Fang Z, Yang Q, Dong H, Lu Y, Lei C, et al SirT1 mediates hyperbaric oxygen preconditioning-induced ischemic tolerance in rat brain. Journal of cerebral blood flow and metabolism. 2013;33(3):396–406. 10.1038/jcbfm.2012.179 23299244 PMC3587810

[40] Liu JR, Zhao Y, Patzer A, Staak N, Boehm R, Deuschl G, et al The c-Jun N-terminal kinase (JNK) inhibitor XG-102 enhances the neuroprotection of hyperbaric oxygen after cerebral ischaemia in adult rats. Neuropathology and applied neurobiology. 2010;36(3):211–24. 10.1111/j.1365-2990.2009.01047.x 19849792

[41] Jiang Q, Huo B. The effect of hyperbaric oxygen on infarct volume and the bcl-2 protein in brain after focal cerebral ischemia in rats. Chinese Journal of Rehabilitation Medicine. 2006;21(10):890–2.

[42] Hou H, Grinberg O, Williams B, Grinberg S, Yu H, Alvarenga DL, et al The effect of oxygen therapy on brain damage and cerebral pO(2) in transient focal cerebral ischemia in the rat. Physiological measurement. 2007;28(8):963–76. 17664686 10.1088/0967-3334/28/8/017

[43] Huang ZX, Kang ZM, Gu GJ, Peng GN, Yun L, Tao HY, et al Therapeutic effects of hyperbaric oxygen in a rat model of endothelin-1-induced focal cerebral ischemia. Brain research. 2007;1153:204–13. 17462608 10.1016/j.brainres.2007.03.061

[44] Kawamura S, Yasui N, Shirasawa M, Fukasawa H. Therapeutic effects of hyperbaric oxygenation on acute focal cerebral ischemia in rats. Surgical neurology. 1990;34(2):101–6. 2367929 10.1016/0090-3019(90)90104-w

[45] Lou M, Eschenfelder CC, Herdegen T, Brecht S, Deuschl G. Therapeutic window for use of hyperbaric oxygenation in focal transient ischemia in rats. Stroke. 2004;35(2):578–83. 14715976 10.1161/01.STR.0000111599.77426.A0

[46] Yin D, Zhang JH. Delayed and multiple hyperbaric oxygen treatments expand therapeutic window in rat focal cerebral ischemic model. Neurocritical care. 2005;2(2):206–11. 16159067 10.1385/NCC:2:2:206

[47] Xue L, Yu Q, Zhang H, Liu Y, Wang C, Wang Y. Effect of large dose hyperbaric oxygenation therapy on prognosis and oxidative stress of acute permanent cerebral ischemic stroke in rats. Neurological research. 2008;30(4):389–93. 10.1179/174313208X300413 18544257

[48] Xiong L, Zhu Z, Dong H, Hu W, Hou L, Chen S. Hyperbaric oxygen preconditioning induces neuroprotection against ischemia in transient not permanent middle cerebral artery occlusion rat model. Chinese medical journal. 2000;113(9):836–9. 11776082

[49] Sunami K, Takeda Y, Hashimoto M, Hirakawa M. Hyperbaric oxygen reduces infarct volume in rats by increasing oxygen supply to the ischemic periphery. Critical care medicine. 2000;28(8):2831–6. 10966258 10.1097/00003246-200008000-00025

[50] Harman F, Hasturk AE, Duz B, Gonul E, Korkmaz A. An evaluation of the effectiveness of pre-ischemic hyperbaric oxygen and post-ischemic aminoguanidine in experimental cerebral ischemia. Neurosciences. 2012;17(2):121–6. 22465885

[51] Acka G, Sen A, Canakci Z, Yildiz S, Akin A, Uzun G, et al Effect of combined therapy with hyperbaric oxygen and antioxidant on infarct volume after permanent focal cerebral ischemia. Physiological research. 2007;56(3):369–73. 16792474 10.33549/physiolres.930907

[52] Yin W, Badr AE, Mychaskiw G, Zhang JH. Down regulation of COX-2 is involved in hyperbaric oxygen treatment in a rat transient focal cerebral ischemia model. Brain research. 2002;926(1–2):165–71. 11814419 10.1016/s0006-8993(01)03304-2

[53] Badr AE, Yin W, Mychaskiw G, Zhang JH. Effect of hyperbaric oxygen on striatal metabolites: a microdialysis study in awake freely moving rats after MCA occlusion. Brain research. 2001;916(1–2):85–90. 11597594 10.1016/s0006-8993(01)02867-0

[54] Veltkamp R, Siebing DA, Heiland S, Schoenffeldt-Varas P, Veltkamp C, Schwaninger M, et al Hyperbaric oxygen induces rapid protection against focal cerebral ischemia. Brain research. 2005;1037(1–2):134–8. 15777761 10.1016/j.brainres.2005.01.006

[55] Li J, Liu W, Ding S, Xu W, Guan Y, Zhang JH, et al Hyperbaric oxygen preconditioning induces tolerance against brain ischemia-reperfusion injury by upregulation of antioxidant enzymes in rats. Brain research. 2008;1210:223–9. 10.1016/j.brainres.2008.03.007 18407255

[56] Veltkamp R, Siebing DA, Sun L, Heiland S, Bieber K, Marti HH, et al Hyperbaric oxygen reduces blood-brain barrier damage and edema after transient focal cerebral ischemia. Stroke. 2005;36(8):1679–83. 16020761 10.1161/01.STR.0000173408.94728.79

[57] Sun L, Marti HH, Veltkamp R. Hyperbaric oxygen reduces tissue hypoxia and hypoxia-inducible factor-1 alpha expression in focal cerebral ischemia. Stroke. 2008;39(3):1000–6. 10.1161/STROKEAHA.107.490599 18239183

[58] Lou M, Zhang H, Wang J, Wen SQ, Tang ZQ, Chen YZ, et al Hyperbaric oxygen treatment attenuated the decrease in regional glucose metabolism of rats subjected to focal cerebral ischemia: a high resolution positron emission tomography study. Neuroscience. 2007;146(2):555–61. 17367940 10.1016/j.neuroscience.2007.01.046

[59] Henninger N, Kuppers-Tiedt L, Sicard KM, Gunther A, Schneider D, Schwab S. Neuroprotective effect of hyperbaric oxygen therapy monitored by MR-imaging after embolic stroke in rats. Experimental neurology. 2006;201(2):316–23. 16814772 10.1016/j.expneurol.2006.04.011

[60] Yang ZJ, Xie Y, Bosco GM, Chen C, Camporesi EM. Hyperbaric oxygenation alleviates MCAO-induced brain injury and reduces hydroxyl radical formation and glutamate release. Eur J Appl Physiol. 2010;108(3):513–22. 10.1007/s00421-009-1229-9 19851780

[61] Lei X, Chao H, Zhang Z, Lv J, Li S, Wei H, et al Neuroprotective effects of quercetin in a mouse model of brain ischemic/reperfusion injury via antiapoptotic mechanisms based on the Akt pathway. Molecular medicine reports. 2015;12(3):3688–96. 10.3892/mmr.2015.3857 26016839

[62] Paciaroni M, Caso V, Agnelli G. The concept of ischemic penumbra in acute stroke and therapeutic opportunities. European neurology. 2009;61(6):321–30. 10.1159/000210544 19365124

[63] Liu R, Yang SH. Window of opportunity: estrogen as a treatment for ischemic stroke. Brain research. 2013;1514:83–90. 10.1016/j.brainres.2013.01.023 23340160 PMC3664650

[64] Korkmaz A, Oter S, Sadir S, Topal T, Uysal B, Ozler M, et al Exposure time related oxidative action of hyperbaric oxygen in rat brain. Neurochemical research. 2008;33(1):160–6. 17710543 10.1007/s11064-007-9428-4

[65] Zhou JG, Fang YQ, Liu CY, Zhou YQ, Ji YF, Liu JC. Effect of hyperbaric oxygen on the expression of nitric oxide synthase mRNA in cortex after acute traumatic cerebral injury. Zhongguo ying yong sheng li xue za zhi. 2012;28(1):38–41. 22493892

[66] Anderson DC, Bottini AG, Jagiella WM, Westphal B, Ford S, Rockswold GL, et al A pilot study of hyperbaric oxygen in the treatment of human stroke. Stroke. 1991;22(9):1137–42. 1926256 10.1161/01.str.22.9.1137

[67] Nighoghossian N, Trouillas P, Adeleine P, Salord F. Hyperbaric oxygen in the treatment of acute ischemic stroke. A double-blind pilot study. Stroke. 1995;26(8):1369–72. 7631339 10.1161/01.str.26.8.1369

[68] Imai K, Mori T, Izumoto H, Takabatake N, Kunieda T, Watanabe M. Hyperbaric oxygen combined with intravenous edaravone for treatment of acute embolic stroke: a pilot clinical trial. Neurologia medico-chirurgica. 2006;46(8):373–8. 16936457 10.2176/nmc.46.373

[69] Huang KL, Wu JN, Lin HC, Mao SP, Kang B, Wan FJ. Prolonged exposure to hyperbaric oxygen induces neuronal damage in primary rat cortical cultures. Neuroscience letters. 2000;293(3):159–62. 11036185 10.1016/s0304-3940(00)01493-2

[70] Chavko M, Xing G, Keyser DO. Increased sensitivity to seizures in repeated exposures to hyperbaric oxygen: role of NOS activation. Brain research. 2001;900(2):227–33. 11334802 10.1016/s0006-8993(01)02301-0

[71] Pablos MI, Reiter RJ, Chuang JI, Ortiz GG, Guerrero JM, Sewerynek E, et al Acutely administered melatonin reduces oxidative damage in lung and brain induced by hyperbaric oxygen. Journal of applied physiology. 1997;83(2):354–8. 9262426 10.1152/jappl.1997.83.2.354

[72] Demchenko IT, Boso AE, Bennett PB, Whorton AR, Piantadosi CA. Hyperbaric oxygen reduces cerebral blood flow by inactivating nitric oxide. Nitric oxide. 2000;4(6):597–608. 11139368 10.1006/niox.2000.0313

