# Supplementary figures and images for: The Efficacy of Hyperbaric Oxygen Therapy on Middle Cerebral Artery Occlusion in Animal Studies: A Meta-Analysis
Source: PLoS One. 2016 Feb 9;11(2):e0148324. doi: 10.1371/journal.pone.0148324 (PMC4747521; doi:10.1371/journal.pone.0148324)

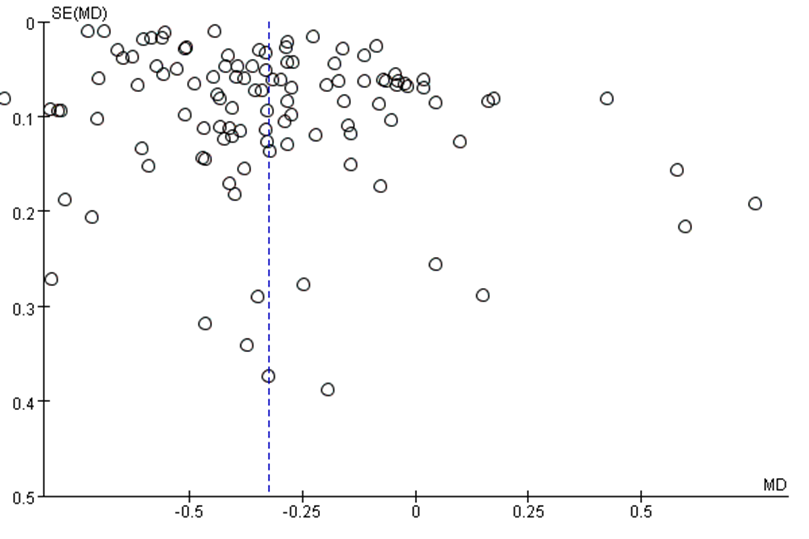

Supplement: S1 Fig — (TIF) [file pone.0148324.s002.tif]
